# Supplementary material for: Association of Plasma Lipopolysaccharide-Binding Protein Concentration with Dietary Factors, Gut Microbiota, and Health Status in the Japanese General Adult Population: A Cross-Sectional Study
Source: Metabolites. 2023 Feb 9;13(2):250. doi: 10.3390/metabo13020250 (PMC9965710; doi:10.3390/metabo13020250)
Supplement: Supplementary file 1 [file metabolites-13-00250-s001.zip › metabolites-2155230-supplementary.pdf]

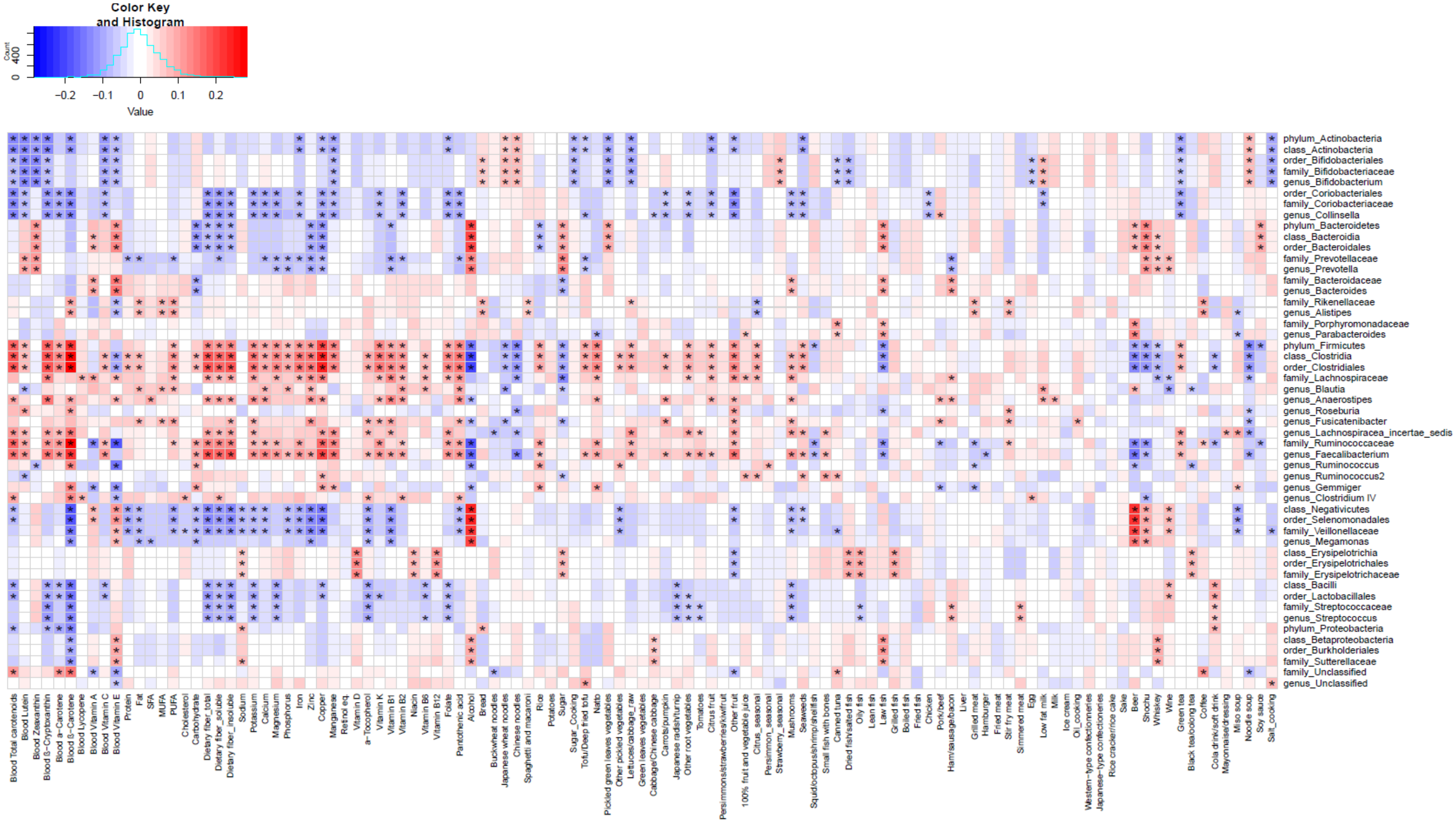

Figure S1. Heatmap showing the relationship between dietary factors and gut microbiota. The positive correlation is shown in red, and the negative correlation is shown in blue. The color intensity shows the magnitude of β values. False discovery rates were evaluated by Storey method. * $Q < 0.20$.

Table S1. Measurement methods of clinical markers and clinical scores.

Category	Variable	Method
Obesity	Abdominal circumference (cm)	Measured using a measuring tape
	Body fat percentage (%)	Body composition analyzer (Tanita MC190, TANITA Corp., Tokyo, Japan)
	Body water content (kg)	Body composition analyzer (Tanita MC190, TANITA Corp.)
	Visceral fat level (levels)	Body composition analyzer (Tanita MC190, TANITA Corp.); Visceral fat levels were determined by firmware embedded in the body composition analyser using a proprietary equation developed by the manufacturer. Levels 9 and below are standard, levels 10-14 are slightly excessive and levels 15 and above indicate excessive visceral fat accumulation. Level 10 corresponds to 100 cm^2 of visceral fat measured on a computerized tomography scan.
	Basal metabolic rate (kcal/day)	Body composition analyzer (Tanita MC190, TANITA Corp.)
	Basal metabolic rate level (levels)	Body composition analyzer (Tanita MC190, TANITA Corp.); Basal metabolic rate levels were determined by the firmware built into the body composition analyser using a unique formula developed by the manufacturer. Specifically, basal metabolic rate levels were calculated on a scale of 1-16 based on the average and statistical distribution of basal metabolic reference values by age group. Levels 6 and below indicated low fat burning, levels 7-10 were standard and levels 11 and above indicated easy burning.
Blood pressure	Systolic blood pressure (mmHg)	Sphygmomanometer (HEM-1040, OMRON Corp., Kyoto, Japan; or ES-P2000BR, Terumo Corp., Tokyo, Japan)
	Diastolic blood pressure (mmHg)	Sphygmomanometer (HEM-1040, OMRON Corp.; or ES-P2000BR, Terumo Corp.)
Arteriosclerosis	baPWV (cm/s)	Network arterial stiffness detection device (BP-203RPEⅢ, OMRON Corp.)
Glucose metabolism	HOMA-IR	Calculated from blood glucose and insulin levels as previously described [130].
	Blood glucose (mg/dL)	Glucokinase-glucose-6-phosphate dehydrogenase method (measurements were performed by LSI Medience Corp. [Tokyo, Japan], a clinical laboratory company)
	HbA1c (%)	Enzymatic method (measurements were performed by LSI Medience Corp.)

	Blood insulin (μU/mL)	Chemiluminescent immunoassay (measurements were performed by LSI Medience Corp.)
	C peptide (ng/mL)	Chemiluminescent immunoassay (measurements were performed by LSI Medience Corp.)
	Glycoalbumin (%)	Enzymatic method (measurements were performed by LSI Medience Corp.)
Lipid metabolism	Triglyceride (mg/dL)	Enzymatic method (measurements were performed by LSI Medience Corp.)
	Total cholesterol (mg/dL)	Enzymatic method (measurements were performed by LSI Medience Corp.)
	HDL-cholesterol (mg/dL)	Enzymatic method (measurements were performed by LSI Medience Corp.)
	LDL-cholesterol (mg/dL)	Enzymatic method (measurements were performed by LSI Medience Corp.)
Physical function	Estimated bone mass (kg)	Body composition analyzer (Tanita MC190, TANITA Corp., Tokyo, Japan)
	Muscle mass (kg)	Body composition analyzer (Tanita MC190, TANITA Corp.)
	Right leg (kg)	Body composition analyzer (Tanita MC190, TANITA Corp.)
	Left leg (kg)	Body composition analyzer (Tanita MC190, TANITA Corp.)
	Right arm (kg)	Body composition analyzer (Tanita MC190, TANITA Corp.)
	Left arm (kg)	Body composition analyzer (Tanita MC190, TANITA Corp.)
	Trunk (kg)	Body composition analyzer (Tanita MC190, TANITA Corp.)
	Locomo 25 score (points)	Locomo 25 score was evaluated with Locomo 25 questionnaire, a screening tool for locomotive syndrome established by a Japanese orthopedic surgeon group [131]. It consists of 25 questions aimed at musculoskeletal disorders such as walking disability, difficulty in daily living, or suffering pain within the body. Scores can range from 0 to 100, and higher scores indicate a greater decline of mobility function.
Eyesight	Far-sightedness	Automatic visual analyzer (CA-1000, TOMEY CORP., Nagoya, Japan); Far-sightedness was determined with Landolt ring as previously described [132].
	Near-sightedness	Automatic visual analyzer (CA-1000, TOMEY CORP.); Near-sightedness was determined with Landolt ring as previously described [132].
Liver function	Albumin (g/dL)	Bromcresol purple method (measurements were performed by LSI Medience Corp.)

	AST (U/L)	Japan Society of Clinical Chemistry transferable method (measurements were performed by LSI Medience Corp.)
	ALT (U/L)	Japan Society of Clinical Chemistry transferable method (measurements were performed by LSI Medience Corp.)
	γ-GTP (U/L)	Japan Society of Clinical Chemistry transferable method (measurements were performed by LSI Medience Corp.)
	Total bilirubin (mg/dL)	Enzymatic method (measurements were performed by LSI Medience Corp.)
	Total Protein (g/dL)	Burette method (measurements were performed by LSI Medience Corp.)
Renal function	Creatinine (mg/dL)	Enzymatic method (measurements were performed by LSI Medience Corp.)
	Blood urea nitrogen (mg/dL)	Urease-glutamate dehydrogenase method (measurements were performed by LSI Medience Corp.)
	Plasma renin activity (ng/mL/hr)	Enzyme immunoassay (measurements were performed by LSI Medience Corp.)
	Urine albumin creatinine ratio (mg/gCr)	Turbidimetric immunoassay (measurements were performed by LSI Medience Corp.)
Adrenal cortex function	Aldosterone (pg/mL)	Radioimmunoassay (measurements were performed by LSI Medience Corp.)
	Cortisol (μg/dL)	Chemiluminescent immunoassay (measurements were performed by LSI Medience Corp.)
Cardiac function	Brain natriuretic peptide (pg/mL)	Chemiluminescent immunoassay (measurements were performed by LSI Medience Corp.)
	Troponin I (pg/mL)	Chemiluminescent immunoassay (measurements were performed by LSI Medience Corp.)
Thyroid function	Free thyroxine (ng/dL)	Electro Chemiluminescence Immunoassay (measurements were performed by LSI Medience Corp.)
	Thyroid stimulating hormone (μIU/mL)	Chemiluminescent immunoassay (measurements were performed by LSI Medience Corp.)
Genital function	Testosterone (ng/mL)	Chemiluminescent immunoassay (measurements were performed by LSI Medience Corp.)
Inflammation	Neutrophil (%)	Flow cytometry (measurements were performed by LSI Medience Corp.)

	Stab neutrophil (%)	Flow cytometry (measurements were performed by LSI Medience Corp.)
	Segmented neutrophil (%)	Flow cytometry (measurements were performed by LSI Medience Corp.)
	Lymphocyte (%)	Flow cytometry (measurements were performed by LSI Medience Corp.)
	Monocyte (%)	Flow cytometry (measurements were performed by LSI Medience Corp.)
	Eosinophil (%)	Flow cytometry (measurements were performed by LSI Medience Corp.)
	Basophil (%)	Flow cytometry (measurements were performed by LSI Medience Corp.)
	IgG (mg/dL)	Turbidimetric immunoassay (measurements were performed by LSI Medience Corp.)
	IgA (mg/dL)	Turbidimetric immunoassay (measurements were performed by LSI Medience Corp.)
	IgM (mg/dL)	Turbidimetric immunoassay (measurements were performed by LSI Medience Corp.)
	Complement C3 (mg/dL)	Turbidimetric immunoassay (measurements were performed by LSI Medience Corp.)
	Complement C4 (mg/dL)	Turbidimetric immunoassay (measurements were performed by LSI Medience Corp.)
	hs-CRP (mg/dL)	Nephelometric method (measurements were performed by LSI Medience Corp.)
	IL-6 (pg/mL)	Chemiluminescence enzyme immunoassay (measurements were performed by LSI Medience Corp.)
Oxidative stress	8-OHdG (ng/mL)	Enzyme immunoassay (measurements were performed by LSI Medience Corp.)
Hematological test	White blood cell (cells/μL)	Flow cytometry (measurements were performed by LSI Medience Corp.)
	Red blood cell (10^4 cells/μL)	Electrical resistance detection (measurements were performed by LSI Medience Corp.)
	Hemoglobin (g/dL)	Sodium Lauryl Sulfate-hemoglobin method (measurements were performed by LSI Medience Corp.)
	Hematocrit (%)	Erythrocyte pulse wave height detection method (measurements were performed by LSI Medience Corp.)

	Mean cell volume (fL)	Electrical resistance detection (measurements were performed by LSI Medience Corp.)
	Mean corpuscular hemoglobin (pg)	Electrical resistance detection (measurements were performed by LSI Medience Corp.)
	Mean cell hemoglobin concentration (g/dL)	Electrical resistance detection (measurements were performed by LSI Medience Corp.)
	Platelet (10^4 cells/μL)	Electrical resistance detection (measurements were performed by LSI Medience Corp.)
Iron metabolism	Ferritin (ng/mL)	Chemiluminescence enzyme immunoassay (measurements were performed by LSI Medience Corp.)
	Serum iron (mmol/L)	Colorimetric method (measurements were performed by LSI Medience Corp.)
Electrolyte	Blood sodium (mmol/L)	Electrode method (measurements were performed by LSI Medience Corp.)
	Blood potassium (mmol/L)	Electrode method (measurements were performed by LSI Medience Corp.)
Allergy	Total IgE (IU/mL)	Fluorescence enzyme immunoassay (measurements were performed by LSI Medience Corp.)
	Anti-poaceae pollen IgE (UA/mL)	Fluorescence enzyme immunoassay (measurements were performed by LSI Medience Corp.)
	Anti-Weed IgE (UA/mL)	Fluorescence enzyme immunoassay (measurements were performed by LSI Medience Corp.)
	Anti-House dust IgE (UA/mL)	Fluorescence enzyme immunoassay (measurements were performed by LSI Medience Corp.)
	Anti-Cedar pollen IgE (UA/mL)	Fluorescence enzyme immunoassay (measurements were performed by LSI Medience Corp.)
Rheumatoid arthritis	Anti-CCP antibody (U/mL)	Fluorescence enzyme immunoassay (measurements were performed by LSI Medience Corp.)
Pituitary gland function	Prolactin (ng/mL)	Chemiluminescence enzyme immunoassay (measurements were performed by LSI Medience Corp.)
	Follicle stimulating hormone (mlU/mL)	Chemiluminescence enzyme immunoassay (measurements were performed by LSI Medience Corp.)

	Adrenocorticotropic hormone (pg/mL)	Electrochemiluminescence immunoassay (measurements were performed by LSI Medience Corp.)
Asthma/ airway inflammation	FVC (L)	Spirometer (HI-801, CHEST M.I., Inc., Tokyo, Japan)
	FVC to predicted FVC ratio (%)	Spirometer (HI-801, CHEST M.I., Inc.)
	FEV1 (L)	Spirometer (HI-801, CHEST M.I., Inc.)
	FEV1 to predicted FEV1 ratio (%)	Spirometer (HI-801, CHEST M.I., Inc.)
	FEV1%G (%)	Spirometer (HI-801, CHEST M.I., Inc.)
	FEV1%G to predicted FEV1%G ratio (%)	Spirometer (HI-801, CHEST M.I., Inc.)
	MMF (L/s)	Spirometer (HI-801, CHEST M.I., Inc.)
	MMF to predicted MMF ratio (%)	Spirometer (HI-801, CHEST M.I., Inc.)
	PEF (L/s)	Spirometer (HI-801, CHEST M.I., Inc.)
	PEF to predicted PEF ratio (%)	Spirometer (HI-801, CHEST M.I., Inc.)
	PEF time (seconds)	Spirometer (HI-801, CHEST M.I., Inc.)
	FET (seconds)	Spirometer (HI-801, CHEST M.I., Inc.)
	EV (%)	Spirometer (HI-801, CHEST M.I., Inc.)
	Exhaled H_2 (ppm)	TRI lyser mBA-3000 (measurements were performed by TAIYO Instruments INC. [Osaka, Japan])
	Exhaled CH_4 (ppm)	TRI lyser mBA-3000 (measurements were performed by TAIYO Instruments INC.)
Mental health	S-WHO-5 score (points)	The S-WHO-5 score was evaluated with the questionnaire. It is a modified version of the WHO-5, a tool for assessing mental health status, established by Inagaki et al [133]. Specifically, the WHO-5 uses a 6-point scale to answer each question, while the S-WHO-5 uses a 4-point scale. Scores range from 0 to 15 points, with higher scores indicating better mental health.

Depression	CES-D score (points)	CES-D score was evaluated with the questionnaire, established by Radloff LS [134]. Scores range from 0 to 60 points, with higher scores indicating sever depressive symptoms.
Cognitive function	MMSE score (points)	MMSE score was evaluated with the questionnaire, established by Folstein et. al [135]. Scores range from 0 to 30 points, with lower scores indicating decline of cognitive functions.

baPWV, brachial-ankle pulse wave velocity; HOMA-IR, homeostasis model assessment-insulin resistance; HbA1c, Hemoglobin A1c; HDL, high-density lipoprotein; LDL, low-density lipoprotein; AST, aspartate aminotransferase; ALT, alanine aminotransferase; γ-GTP, γ-glutamyltransferase; Ig, immunoglobulin; hs-CRP, high-sensitivity C-reactive protein; IL, interleukin; 8-OHdG, 8-hydroxy-2-deoxyguanosine; CCP, cyclic citrullinated peptide; FVC, forced vital capacity; FEV, forced expiratory volume in one second; FEV1%G; FEV1/FVC; MMF, maximum mid-expiratory flow; PEF, peak expiratory flow; FET, Forced expiration time; EV, extrapolated volume; S-WHO-5, simplified Japanese version of the World Health Organization-Five Well-Being Index; CES-D, Center for Epidemiological Studies Depression; MMSE, Mini-Mental state examination

Table S2. Values of clinical markers and clinical scores of the participants.

Category	Variable	All subjects			Male			Female		
		Mean ± SD	Median	IQR	Mean ± SD	Median	IQR	Mean ± SD	Median	IQR
Obesity	Body fat percentage (%)	26.1 ± 8.1	25.5	20.3 - 31.9	20.6 ± 5.8	20.5	16.8 - 23.7	30.2 ± 7.1	30.2	25.4 - 34.7
	Body water content (kg)	31.4 ± 6.3	29.8	26.4 - 35.6	37.2 ± 5.0	36.9	33.9 - 40.0	27.2 ± 3.0	27.0	25.1 - 29.1
	Visceral fat level (levels)	8.1 ± 4.3	8.0	5.0 - 11.0	11.3 ± 4.0	11.0	9.0 - 14.0	5.9 ± 2.9	6.0	4.0 - 8.0
	Basal metabolic rate (kcal/day)	1260 ± 247	1190	1060 - 1420	1477 ± 205	1456	1339 - 1589	1097 ± 120	1084	1016 - 1162
	Basal metabolic rate level (levels)	10.0 ± 3.3	9.0	8.0 - 13.0	10.4 ± 3.0	10.0	9.0 - 13.0	9.6 ± 3.4	9.0	7.0 - 12.0
Glucose metabolism	C peptide (ng/mL)	1.3 ± 0.6	1.2	0.9 - 1.6	1.4 ± 0.6	1.3	1.0 - 1.7	1.3 ± 0.5	1.2	0.9 - 1.5
	Glycoalbumin (%)	14.4 ± 1.8	14.2	13.3 - 15.1	14.1 ± 2.2	13.7	12.9 - 14.8	14.6 ± 1.4	14.4	13.8 - 15.2
Physical function	Estimated bone mass (kg)	2.4 ± 0.4	2.4	2.1 - 2.8	2.8 ± 0.3	2.8	2.6 - 3.0	2.1 ± 0.3	2.1	1.9 - 2.3
	Mascle mass (kg)	41.9 ± 8.9	38.7	34.7 - 49.2	50.9 ± 6.0	50.4	47.0 - 54.4	35.4 ± 3.1	35.3	33.3 - 37.4
	Right leg (kg)	7.4 ± 1.9	6.9	6.0 - 8.7	9.1 ± 1.5	9.0	8.1 - 10.0	6.2 ± 0.8	6.1	5.7 - 6.7

	Left leg (kg)	7.3 ± 1.8	6.9	5.9 - 8.6	9.0 ± 1.5	8.9	7.9 - 9.9	6.1 ± 0.8	6.1	5.6 - 6.6
	Right arm (kg)	2.1 ± 0.6	1.9	1.7 - 2.6	2.7 ± 0.4	2.7	2.5 - 3.0	1.7 ± 0.2	1.7	1.6 - 1.8
	Left arm (kg)	2.1 ± 0.6	1.8	1.6 - 2.6	2.7 ± 0.4	2.6	2.4 - 2.9	1.6 ± 0.2	1.6	1.5 - 1.8
	Trunk (kg)	23.0 ± 4.3	21.5	19.6 - 26.9	27.4 ± 2.6	27.4	25.8 - 29.1	19.8 ± 1.6	19.9	18.7 - 20.9
	Locomo 25 score (points)	6.9 ± 9.0	4.0	2.0 - 9.0	5.9 ± 7.8	3.0	1.0 - 7.0	7.6 ± 9.7	4.0	2.0 - 9.0
Eyesight	Far-sightedness	1.1 ± 0.3	1.0	0.9 - 1.2	1.1 ± 0.3	1.2	0.9 - 1.5	1.0 ± 0.3	1.0	0.8 - 1.2
	Near-sightedness	0.9 ± 0.4	0.9	0.7 - 1.2	0.9 ± 0.4	0.9	0.6 - 1.2	0.9 ± 0.3	0.9	0.7 - 1.2
Liver function	Albumin (g/dL)	4.5 ± 0.3	4.5	4.3 - 4.6	4.5 ± 0.3	4.5	4.3 - 4.7	4.4 ± 0.3	4.4	4.3 - 4.6
	AST (U/L)	23.4 ± 12.8	22.0	18.0 - 26.0	25.2 ± 9.1	23.0	19.0 - 28.0	22.2 ± 14.9	20.0	17.0 - 24.0
	ALT (U/L)	21.9 ± 15.5	18.0	14.0 - 24.0	26.5 ± 15.0	22.0	18.0 - 31.0	18.6 ± 15.0	16.0	12.0 - 20.0
	γ-GTP (U/L)	34.6 ± 47.1	22.0	15.0 - 38.0	49.6 ± 58.1	35.0	21.0 - 55.0	23.7 ± 33.4	17.0	13.0 - 25.0
	Total bilirubin (mg/dL)	0.8 ± 0.3	0.8	0.6 - 1.0	0.9 ± 0.4	0.8	0.6 - 1.0	0.8 ± 0.3	0.8	0.6 - 1.0
	Total Protein (g/dL)	7.3 ± 0.4	7.3	7.1 - 7.6	7.3 ± 0.4	7.4	7.1 - 7.6	7.3 ± 0.4	7.3	7.1 - 7.6
Renal function	Creatinine (mg/dL)	0.7 ± 0.2	0.7	0.6 - 0.8	0.8 ± 0.1	0.8	0.7 - 0.9	0.6 ± 0.1	0.6	0.6 - 0.7

	Blood urea nitrogen (mg/dL)	14.7 ± 3.9	14.2	12.0 - 17.0	15.4 ± 4.1	14.9	12.6 - 17.7	14.1 ± 3.7	13.7	11.5 - 16.3
	Plasma renin activity (ng/mL/hr)	2.1 ± 4.0	1.0	0.5 - 2.1	2.9 ± 5.6	1.4	0.7 - 2.8	1.4 ± 2.1	0.8	0.4 - 1.7
	Urine albumin creatinine ratio (mg/gCr)	30 ± 149	7	4 - 14	45 ± 216	5	3 - 13	20 ± 67	7	5 - 14
Adrenal cortex function	Aldosterone (pg/mL)	116 ± 50	110	84 - 139	119 ± 46	115	88 - 141	115 ± 53	106	81 - 138
	Cortisol (µg/dL)	8.8 ± 3.1	8.4	6.6 - 10.5	9.6 ± 2.8	9.6	7.6 - 11.3	8.2 ± 3.1	7.6	6.1 - 9.6
Cardiac function	Brain natriuretic peptide (pg/mL)	13.1 ± 15.9	8.2	5.8 - 13.8	11.3 ± 12.7	6.2	5.8 - 11.0	14.4 ± 17.7	9.6	6.0 - 15.4
	Troponin I (pg/mL)	5.9 ± 13.6	4.0	4.0 - 4.0	7.1 ± 20.1	4.0	4.0 - 4.2	5.1 ± 5.2	4.0	4.0 - 4.0
Thyroid function	Free thyroxine (ng/dL)	1.0 ± 0.2	1.0	1.0 - 1.1	1.0 ± 0.1	1.0	1.0 - 1.1	1.0 ± 0.2	1.0	1.0 - 1.1
	Thyroid stimulating hormone (µIU/mL)	1.9 ± 1.7	1.5	1.1 - 2.2	1.8 ± 1.6	1.4	0.9 - 2.1	2.0 ± 1.7	1.6	1.1 - 2.3
Sexual function	Testosterone (ng/mL)	2.8 ± 3.3	0.4	0.2 - 5.6	6.3 ± 2.0	6.2	4.9 - 7.6	0.3 ± 0.1	0.3	0.2 - 0.3
Inflammation	Neutrophil (%)	54.8 ± 8.8	54.5	49.0 - 60.0	55.2 ± 8.7	55.0	49.5 - 60.0	54.5 ± 8.9	54.0	49.0 - 60.0

	Stab neutrophil (%)	2.0 ± 1.5	2.0	1.0 - 2.0	2.1 ± 1.5	2.0	1.0 - 2.8	2.0 ± 1.4	2.0	1.0 - 2.0
	Segmented neutrophil (%)	52.7 ± 8.7	52.0	47.0 - 58.0	53.0 ± 8.6	53.0	47.0 - 58.0	52.5 ± 8.8	52.0	47.0 - 58.0
	Lymphocyte (%)	35.7 ± 8.2	36.0	30.0 - 41.0	34.7 ± 7.9	35.0	29.0 - 40.0	36.4 ± 8.3	36.0	31.0 - 42.0
	Monocyte (%)	5.9 ± 2.0	6.0	4.0 - 7.0	6.2 ± 1.9	6.0	5.0 - 8.0	5.6 ± 2.0	5.5	4.0 - 7.0
	Eosinophil (%)	3.0 ± 2.1	2.5	2.0 - 4.0	3.2 ± 2.1	3.0	2.0 - 4.0	2.8 ± 2.0	2.0	1.0 - 4.0
	Basophil (%)	0.7 ± 0.6	1.0	0.0 - 1.0	0.6 ± 0.6	1.0	0.0 - 1.0	0.7 ± 0.6	1.0	0.0 - 1.0
	IgG (mg/dL)	1220 ± 268	1200	1050 - 1380	1193 ± 270	1169	1004 - 1365	1248 ± 265	1230	1075 - 1386
	IgA (mg/dL)	220 ± 91	208	154 - 270	234 ± 93	229	166 - 282	210 ± 88	199	146 - 260
	IgM (mg/dL)	99 ± 55	88	64 - 122	89 ± 57	78	56 - 106	106 ± 53	96	68 - 131
	Complement C3 (mg/dL)	101 ± 16	100	90 - 112	102 ± 16	100	91 - 113	101 ± 16	100	90 - 111
	Complement C4 (mg/dL)	24.5 ± 6.8	24.0	20.0 - 29.0	24.5 ± 6.5	24.0	20.0 - 28.0	24.5 ± 7.0	24.0	20.0 - 29.0
	hs-CRP (mg/dL)	0.06 ± 0.09	0.03	0.01 - 0.07	0.07 ± 0.08	0.03	0.02 - 0.08	0.06 ± 0.09	0.03	0.01 - 0.06
	IL-6 (pg/mL)	1.5 ± 4.0	0.9	0.6 - 1.3	1.6 ± 3.4	0.9	0.7 - 1.4	1.3 ± 4.4	0.8	0.6 - 1.2
Oxidative stress	8-OHdG (ng/mL)	9.2 ± 4.1	8.6	6.4 - 10.8	8.7 ± 3.6	8.1	6.3 - 10.5	9.5 ± 4.5	8.9	6.6 - 11.1

Hematological test	White blood cell (cells/μL)	5630 ± 1620	5400	4600 - 6400	5973 ± 1590	5700	4900 - 6800	5389 ± 1598	5100	4400 - 6100
	Red blood cell (10^4 cells/μL)	462 ± 41	460	432 - 489	486 ± 38	488	461 - 511	444 ± 33	444	420 - 464
	Hemoglobin (g/dL)	14.1 ± 1.4	14.0	13.1 - 15.1	15.2 ± 1.0	15.2	14.5 - 15.9	13.3 ± 1.0	13.3	12.7 - 14.0
	Hematocrit (%)	44.6 ± 3.8	44.5	42.0 - 47.3	47.5 ± 3.0	47.6	45.6 - 49.4	42.6 ± 2.9	42.6	40.7 - 44.5
	Mean cell volume (fL)	97 ± 5	97	94 - 100	98 ± 4	98	94 - 100	96 ± 5	97	94 - 99
	Mean corpuscular hemoglobin (pg)	30.6 ± 1.9	30.8	29.8 - 31.7	31.3 ± 1.5	31.2	30.3 - 32.2	30.0 ± 2.0	30.4	29.4 - 31.3
	Mean cell hemoglobin concentration (g/dL)	31.6 ± 0.9	31.6	31.0 - 32.1	32.0 ± 0.8	32.0	31.6 - 32.5	31.2 ± 0.9	31.3	30.8 - 31.8
	Platelet (10^4 cells/μL)	25.9 ± 5.7	25.2	21.9 - 29.1	25.5 ± 5.6	24.8	21.6 - 29.0	26.1 ± 5.8	25.3	22.0 - 29.4
Iron metabolism	Ferritin (ng/mL)	97 ± 86	77	38 - 129	145 ± 94	124	88 - 175	62 ± 60	51	21 - 81
	Serum iron (mmol/L)	100 ± 40	96	74 - 123	107 ± 41	100	75 - 133	96 ± 38	93	74 - 117
Electrolyte	Blood sodium (mmol/L)	142 ± 2	142	141 - 143	142 ± 2	142	141 - 143	142 ± 2	142	141 - 143
	Blood potassium (mmol/L)	4.0 ± 0.3	4.0	3.8 - 4.2	4.1 ± 0.3	4.1	3.9 - 4.3	4.0 ± 0.2	4.0	3.8 - 4.1
Allergy	Total IgE (IU/mL)	137 ± 487	48	16 - 120	150 ± 287	55	19 - 145	128 ± 590	41	15 - 110

	Anti-poaceae pollen IgE (UA/mL)	1.8 ± 6.5	0.1	0.1 - 0.2	1.9 ± 6.3	0.1	0.1 - 0.4	1.6 ± 6.7	0.1	0.1 - 0.1
	Anti-Weed IgE (UA/mL)	0.4 ± 1.9	0.1	0.1 - 0.1	0.5 ± 2.5	0.1	0.1 - 0.1	0.4 ± 1.4	0.1	0.1 - 0.1
	Anti-House dust IgE (UA/mL)	1.9 ± 5.9	0.1	0.1 - 0.5	1.8 ± 5.5	0.1	0.1 - 0.6	1.9 ± 6.2	0.1	0.1 - 0.4
	Anti-Cedar pollen IgE (UA/mL)	4.3 ± 11.2	0.1	0.1 - 2.4	4.8 ± 12.5	0.2	0.1 - 2.6	4.0 ± 10.2	0.1	0.1 - 2.2
Rheumatoid arthritis	Anti-CCP antibody (U/mL)	4.7 ± 54.6	0.5	0.5 - 0.5	2.0 ± 24.4	0.5	0.5 - 0.5	6.5 ± 68.5	0.5	0.5 - 0.5
Pituitary gland function	Prolactin (ng/mL)	8.7 ± 10.4	6.6	5.2 - 9.4	7.6 ± 12.6	6.3	4.9 - 8.2	9.5 ± 8.3	7.1	5.4 - 10.5
	Follicle stimulating hormone (mlU/mL)	25.3 ± 26.1	9.7	4.5 - 46.9	8.1 ± 9.4	5.3	3.7 - 9.2	37.6 ± 27.3	40.5	6.3 - 57.6
	Adreno-corticotropic hormone (pg/mL)	23.2 ± 14.0	20.6	14.5 - 29.0	28.7 ± 15.1	25.9	19.2 - 33.9	19.3 ± 11.7	17.3	12.4 - 23.4
Asthma/airway inflammation	FVC (L)	3.4 ± 0.9	3.2	2.7 - 4.0	4.1 ± 0.8	4.1	3.6 - 4.6	2.9 ± 0.5	2.9	2.5 - 3.2
	FVC to predicted FVC ratio (%)	112 ± 16	112	102 - 122	110 ± 16	112	102 - 121	113 ± 15	113	102 - 123

FEV1 (L)	2.7 ± 0.7	2.6	2.2 - 3.2	3.2 ± 0.7	3.2	2.7 - 3.8	2.3 ± 0.5	2.3	2.0 - 2.6
FEV1 to predicted FEV1 ratio (%)	108 ± 17	109	97 - 120	107 ± 16	108	97 - 117	110 ± 17	110	98 - 122
FEV1%G (%)	80.6 ± 7.5	80.9	76.5 - 85.1	79.2 ± 7.7	80.1	75.1 - 84.2	81.6 ± 7.1	81.5	77.5 - 85.8
FEV1%G to predicted FEV1%G ratio (%)	107 ± 10	107	101 - 113	110 ± 11	110	104 - 117	105 ± 8	105	100 - 110
MMF (L/s)	2.8 ± 1.1	2.7	2.0 - 3.5	3.2 ± 1.2	3.2	2.4 - 3.9	2.5 ± 0.9	2.4	1.8 - 3.1
MMF to predicted MMF ratio (%)	87 ± 28	84	68 - 105	82 ± 27	81	64 - 98	90 ± 28	85	70 - 108
PEF (L/s)	6.6 ± 2.1	6.3	5.2 - 7.9	8.2 ± 1.9	8.3	6.9 - 9.5	5.5 ± 1.2	5.6	4.8 - 6.3
PEF to predicted PEF ratio (%)	78.5 ± 17.6	78.9	67.0 - 90.3	83.6 ± 18.6	84.5	70.6 - 95.9	74.8 ± 15.8	75.5	65.0 - 85.8
PEF time (seconds)	0.11 ± 0.06	0.09	0.07 - 0.13	0.10 ± 0.05	0.08	0.06 - 0.12	0.12 ± 0.06	0.10	0.08 - 0.13
FET (seconds)	8.2 ± 4.1	7.8	4.9 - 11.1	8.4 ± 4.1	8.0	5.1 - 11.3	8.0 ± 4.1	7.7	4.6 - 11.0
EV (%)	3.7 ± 1.5	3.4	2.8 - 4.3	3.5 ± 1.3	3.3	2.7 - 4.0	3.9 ± 1.6	3.5	2.9 - 4.4
Exhaled H2 (ppm)	11.7 ± 13.5	6.2	2.6 - 15.3	10.8 ± 11.5	6.2	2.7 - 14.9	12.4 ± 14.7	6.1	2.5 - 16.1

	Exhaled CH4 (ppm)	4.1 ± 8.4	2.2	2.0 - 2.6	3.6 ± 6.0	2.2	2.0 - 2.5	4.4 ± 9.8	2.2	2.0 - 2.6
Mental health	S-WHO-5 score (points)	8.6 ± 2.9	9.0	6.0 - 10.0	8.6 ± 2.9	9.0	6.0 - 10.0	8.6 ± 2.8	9.0	7.0 - 10.0
Depression	CES-D score (points)	9.6 ± 8.0	8.0	3.0 - 14.0	8.8 ± 7.7	8.0	3.0 - 12.0	10.1 ± 8.1	8.0	4.0 - 14.0
Cognitive function	Mini-Mental state examinaton score (points)	29.4 ± 1.2	30.0	29.0 - 30.0	29.3 ± 1.3	30.0	29.0 - 30.0	29.5 ± 1.1	30.0	29.0 - 30.0

baPWV, brachial-ankle pulse wave velocity; HOMA-IR, homeostasis model assessment-insulin resistance; HbA1c, Hemoglobin A1c; HDL, high-density lipoprotein; LDL, low-density lipoprotein; AST, aspartate aminotransferase; ALT, alanine aminotransferase; γ-GTP, γ-glutamyltransferase; Ig, immunoglobulin; hs-CRP, high-sensitivity C-reactive protein; IL, interleukin; 8-OHdG, 8-hydroxy-2-deoxyguanosine; CCP, cyclic citrullinated peptide; FVC, forced vital capacity; FEV, forced expiratory volume in one second; FEV1%G; FEV1/FVC; MMF, maximum mid-expiratory flow; PEF, peak expiratory flow; FET, Forced expiration time; EV, extrapolated volume; S-WHO-5, simplified Japanese version of the World Health Organization-Five Well-Being Index; CES-D, Center for Epidemiological Studies Depression; MMSE, Mini-Mental state examination

Table S3. The blood antioxidant concentration, food intake, and nutrition intake of the participants.

Variable	All subjects			Male			Female		
	Mean ± SD	Median	IQR	Mean ± SD	Median	IQR	Mean ± SD	Median	IQR
**Blood antioxidant**									
Total carotenoids (μg/mL)	1.23 ± 0.61	1.09	0.82 - 1.49	1.02 ± 0.55	0.89	0.69 - 1.16	1.39 ± 0.59	1.28	0.98 - 1.65
Lutein (μg/mL)	0.27 ± 0.13	0.24	0.18 - 0.32	0.25 ± 0.13	0.22	0.17 - 0.30	0.28 ± 0.13	0.25	0.20 - 0.33
Zeaxanthin (μg/mL)	0.05 ± 0.02	0.05	0.04 - 0.06	0.05 ± 0.02	0.05	0.04 - 0.06	0.05 ± 0.02	0.04	0.04 - 0.06
β-Cryptoxanthin (μg/mL)	0.14 ± 0.09	0.12	0.08 - 0.17	0.11 ± 0.07	0.09	0.07 - 0.13	0.16 ± 0.09	0.14	0.10 - 0.19
α-Carotene (μg/mL)	0.14 ± 0.14	0.10	0.07 - 0.16	0.12 ± 0.16	0.08	0.05 - 0.12	0.16 ± 0.13	0.13	0.09 - 0.18
β-Carotene (μg/mL)	0.41 ± 0.32	0.33	0.19 - 0.53	0.27 ± 0.26	0.19	0.12 - 0.33	0.51 ± 0.32	0.41	0.28 - 0.65
Lycopene (μg/mL)	0.23 ± 0.14	0.20	0.13 - 0.29	0.21 ± 0.13	0.19	0.12 - 0.27	0.24 ± 0.15	0.22	0.15 - 0.29
Vitamin A (μg/mL)	0.45 ± 0.15	0.44	0.35 - 0.53	0.53 ± 0.15	0.51	0.43 - 0.63	0.40 ± 0.12	0.38	0.31 - 0.47
Vitamin C (μg/mL)	11.6 ± 4.3	11.1	8.9 - 13.9	10.1 ± 3.9	10.0	7.8 - 12.0	12.7 ± 4.3	12.3	10.1 - 14.9
Vitamin E (μg/mL)	11.9 ± 3.4	11.4	9.9 - 13.3	12.0 ± 3.5	11.4	9.8 - 13.3	11.8 ± 3.3	11.4	9.9 - 13.2
**Energy intake**									

Energy (kcal)	1890 ± 589	1800	1490 - 2210	2197 ± 590	2115	1802 - 2539	1672 ± 481	1626	1367 - 1910
**Nutrition intake**									
Protein (g)	71.7 ± 14.7	71.1	63.2 - 79.1	78.0 ± 27.3	75.1	58.2 - 92.4	67.2 ± 24.9	63.8	50.2 - 78.1
Fat (g)	53.5 ± 12.0	53.6	46.4 - 61.3	57.0 ± 20.4	55.2	43.5 - 68.4	51.1 ± 17.9	49.0	38.8 - 61.4
SFA (g)	14.1 ± 3.8	14.2	11.8 - 16.3	15.0 ± 5.9	14.4	10.9 - 18.5	13.5 ± 5.1	12.9	9.9 - 16.3
MUFA (g)	18.9 ± 4.6	18.9	16.1 - 21.8	20.2 ± 7.4	19.4	15.3 - 24.3	18.0 ± 6.5	17.2	13.6 - 22.0
PUFA (g)	13.4 ± 3.1	13.3	11.4 - 15.3	14.3 ± 5.0	13.5	10.7 - 17.3	12.8 ± 4.5	12.4	9.6 - 15.3
Cholesterol (mg)	380 ± 126	370	295 - 455	410 ± 191	391	272 - 506	358 ± 163	331	238 - 444
Carbohydrate (g)	251 ± 37	254	229 - 275	293 ± 86	285	237 - 338	222 ± 67	217	179 - 257
Total dietary fiber (g)	11.2 ± 3.4	10.9	9.0 - 12.9	11.8 ± 4.9	10.9	8.4 - 14.7	10.7 ± 4.2	10.2	7.8 - 12.9
Soluble (g)	2.8 ± 1.0	2.7	2.2 - 3.3	2.9 ± 1.4	2.7	1.9 - 3.6	2.7 ± 1.1	2.5	1.9 - 3.3
Insoluble (g)	8.0 ± 2.3	7.9	6.5 - 9.2	8.5 ± 3.4	8.0	6.2 - 10.6	7.7 ± 3.0	7.3	5.6 - 9.3
Sodium (mg)	4380 ± 892	4390	3850 - 4890	4942 ± 1579	4713	3801 - 5823	3972 ± 1345	3774	3141 - 4588

Potassium (mg)	2350 ± 636	2280	1970 - 2700	2480 ± 922	2336	1861 - 2962	2250 ± 846	2123	1659 - 2681
Calcium (mg)	505 ± 180	490	391 - 609	522 ± 242	471	352 - 666	493 ± 213	459	346 - 605
Magnesium (mg)	252 ± 54	247	219 - 280	274 ± 91	266	209 - 324	236 ± 85	224	178 - 277
Phosphorus (mg)	1060 ± 227	1050	929 - 1170	1151 ± 408	1088	864 - 1364	997 ± 371	936	746 - 1177
Iron (mg)	7.6 ± 1.9	7.4	6.5 - 8.6	8.1 ± 3.1	7.6	5.9 - 9.9	7.3 ± 2.8	6.7	5.3 - 8.8
Zinc (mg)	8.3 ± 1.3	8.3	7.6 - 8.9	9.1 ± 2.7	8.8	7.2 - 10.8	7.7 ± 2.5	7.3	5.9 - 9.0
Copper (mg)	1.2 ± 0.2	1.2	1.0 - 1.3	1.3 ± 0.4	1.2	1.0 - 1.5	1.1 ± 0.4	1.0	0.8 - 1.3
Manganese (mg)	2.9 ± 0.7	2.8	2.4 - 3.3	3.2 ± 1.1	3.0	2.4 - 3.8	2.7 ± 1.0	2.5	2.0 - 3.3
Retinol eq. (µg RE)	619 ± 337	563	412 - 782	664 ± 405	618	360 - 877	586 ± 370	506	359 - 747
Vitamin D (µg)	15.4 ± 9.6	13.8	9.5 - 19.7	16.2 ± 12.2	12.9	7.8 - 20.7	14.9 ± 10.9	12.5	7.4 - 18.9
α-Tocopherol (mg)	6.9 ± 1.9	6.8	5.8 - 8.0	7.2 ± 2.9	6.7	5.3 - 8.9	6.7 ± 2.6	6.2	4.9 - 8.0
Vitamin K (µg)	315 ± 147	295	212 - 399	323 ± 175	296	195 - 432	310 ± 157	289	187 - 400
Vitamin B_1 (mg)	0.73 ± 0.17	0.71	0.63 - 0.82	0.77 ± 0.28	0.74	0.57 - 0.91	0.69 ± 0.25	0.65	0.52 - 0.82
Vitamin B_2 (mg)	1.3 ± 0.3	1.3	1.1 - 1.4	1.3 ± 0.5	1.3	1.0 - 1.6	1.2 ± 0.4	1.1	0.9 - 1.4
Niacin (mg)	17.8 ± 4.9	17.2	14.8 - 20.6	19.7 ± 7.6	18.8	14.8 - 23.5	16.5 ± 6.9	15.2	11.9 - 19.7

Vitamin B_6 (mg)	1.2 ± 0.3	1.2	1.1 - 1.4	1.4 ± 0.5	1.3	1.0 - 1.6	1.2 ± 0.5	1.1	0.8 - 1.4
Vitamin B_{12} (μg)	10.6 ± 5.8	9.9	7.1 - 13.2	11.4 ± 7.6	9.5	6.3 - 14.5	10.0 ± 6.8	8.4	5.5 - 12.5
Folate (μg)	292 ± 99	278	228 - 340	307 ± 130	291	217 - 373	281 ± 119	259	200 - 348
Pantothenic acid (mg)	6.5 ± 1.3	6.5	5.7 - 7.3	7.0 ± 2.3	6.8	5.4 - 8.3	6.1 ± 2.1	5.8	4.6 - 7.2
Alcohol (g)	12.5 ± 19.9	7.6	1.6 - 16.6	23.3 ± 27.0	16.6	0.9 - 35.1	4.8 ± 11.4	0.0	0.0 - 3.8
**Food intake**									
**Cereals**									
Bread (g)	33.2 ± 28.0	26.0	13.0 - 49.9	35.2 ± 32.8	28.9	10.4 - 57.7	31.7 ± 26.0	22.5	10.0 - 50.0
Buckwheat noodles (g)	21.9 ± 25.2	16.5	7.6 - 28.2	28.7 ± 33.2	20.8	9.7 - 52.0	17.1 ± 21.2	9.3	7.5 - 20.0
Japanese wheat noodles (g)	20.4 ± 21.3	14.9	8.2 - 23.8	24.4 ± 25.6	20.8	9.7 - 25.4	17.4 ± 19.1	9.3	7.5 - 20.0
Chinese noodles (g)	24.4 ± 26.2	17.5	9.6 - 29.5	36.0 ± 33.6	23.1	11.9 - 57.7	16.0 ± 18.6	9.3	7.5 - 20.0
Spaghetti and macaroni (g)	12.9 ± 13.8	11.2	4.7 - 17.7	14.9 ± 16.8	10.8	0.0 - 23.1	11.4 ± 13.3	8.4	0.0 - 18.0

Rice (g)	323 ± 122	321	244 - 404	388 ± 164	405	300 - 450	276 ± 116	260	208 - 351
**Potatoes**									
Potatoes (g)	34.0 ± 28.7	25.0	15.1 - 51.3	35.5 ± 33.4	23.1	11.9 - 57.7	32.8 ± 28.0	20.0	10.3 - 50.0
**Sugars and sweeteners**									
Sugar (g)	2.5 ± 4.2	0.5	0.0 - 3.8	3.0 ± 4.7	0.0	0.0 - 5.5	2.1 ± 3.8	0.0	0.0 - 2.8
Cooking sugar (g)	3.2 ± 1.9	2.9	1.6 - 4.5	3.4 ± 2.2	3.2	1.5 - 5.0	3.0 ± 1.8	2.8	1.5 - 4.3
**Pulses**									
Tofu/Deep fried tofu (g)	46.6 ± 35.0	38.0	21.1 - 67.8	42.3 ± 33.8	36.3	16.1 - 48.4	49.6 ± 37.4	34.9	31.4 - 69.8
Natto (g)	21.5 ± 17.2	17.4	8.4 - 34.7	22.4 ± 19.2	18.6	7.4 - 37.1	20.9 ± 17.0	16.1	5.8 - 35.4
**Vegetables**									
Pickled green leaves vegetables (g)	7.7 ± 9.3	5.0	1.8 - 10.8	8.6 ± 10.4	4.2	1.6 - 10.4	7.0 ± 9.2	3.3	0.0 - 9.0
Other pickled vegetables (g)	8.2 ± 10.5	4.8	1.9 - 10.7	8.8 ± 11.5	3.8	1.6 - 10.4	7.7 ± 10.4	3.3	1.4 - 9.0

Raw lettuces/cabbage (g)	25.8 ± 18.9	21.4	12.4 - 35.7	26.2 ± 20.1	20.9	8.3 - 37.9	25.6 ± 19.3	18.1	14.8 - 36.1
Green leaves vegetables (g)	30.4 ± 30.8	25.2	11.1 - 34.8	29.0 ± 32.8	14.6	6.8 - 33.2	31.4 ± 30.0	25.9	11.5 - 34.5
Cabbage/Chinese cabbage (g)	31.0 ± 23.9	27.9	15.2 - 36.8	31.1 ± 26.2	29.9	13.3 - 36.5	30.9 ± 24.5	25.9	11.5 - 34.5
Carrots/pumpkin (g)	16.3 ± 13.8	15.0	6.7 - 19.6	14.4 ± 13.7	8.3	3.9 - 19.0	17.6 ± 14.3	14.8	6.6 - 19.7
Japanese radish/turnip (g)	14.7 ± 16.4	10.7	5.9 - 21.6	14.9 ± 17.0	11.4	5.3 - 25.6	14.6 ± 17.3	8.9	4.6 - 22.2
Other root vegetables (g)	29.9 ± 23.5	25.3	14.4 - 40.8	28.5 ± 25.6	25.6	10.2 - 31.3	30.9 ± 23.6	24.6	19.7 - 44.4
Tomatoes (g)	23.9 ± 24.7	15.7	7.8 - 30.3	23.2 ± 25.9	12.5	5.3 - 31.3	24.4 ± 25.0	19.7	5.1 - 29.6
**Fruits**									
Citrus fruit (g)	10.6 ± 18.1	6.0	1.0 - 12.9	9.9 ± 18.8	6.9	0.0 - 14.8	11.1 ± 17.8	6.0	0.0 - 12.9
Persimmons/ strawberries/ kiwifruit (g)	8.3 ± 14.2	6.0	0.1 - 7.1	6.4 ± 11.2	0.0	0.0 - 6.9	9.7 ± 15.8	6.0	0.0 - 12.9

Other fruit (g)	35.1 ± 38.8	20.0	8.5 - 55.0	33.7 ± 39.4	14.8	6.9 - 37.1	36.1 ± 39.6	32.1	6.0 - 64.3
100% fruit and vegetable juice (g)	38.9 ± 68.9	17.3	3.4 - 47.9	49.6 ± 89.9	15.4	0.0 - 82.5	31.3 ± 51.7	13.3	0.0 - 28.6
Seasonal citrus (g)	11.2 ± 12.2	8.2	2.2 - 16.6	9.6 ± 11.6	3.7	1.7 - 9.3	12.4 ± 12.4	8.0	3.2 - 16.1
Seasonal persimmon (g)	6.3 ± 10.3	2.0	0.3 - 8.4	4.8 ± 9.6	1.7	0.0 - 3.7	7.4 ± 10.7	1.5	0.0 - 8.0
Seasonal strawberry (g)	7.5 ± 10.5	3.2	2.2 - 12.1	6.2 ± 9.5	2.7	2.7 - 5.8	8.5 ± 11.1	5.0	2.3 - 12.5
**Mushroom**									
Mushrooms (g)	10.4 ± 9.0	9.6	4.3 - 12.4	9.3 ± 8.1	5.2	4.0 - 11.9	11.3 ± 9.8	9.2	4.1 - 12.3
**Algae**									
Seaweeds (g)	11.7 ± 10.8	8.9	4.9 - 14.7	12.0 ± 12.3	6.3	3.2 - 15.7	11.4 ± 10.7	11.1	4.4 - 13.6
**Fish and shellfish**									

Squid/ octopus/ shrimp/ shellfish (g)	14.4 ± 15.2	11.2	6.6 - 17.1	16.8 ± 16.0	13.7	7.1 - 16.7	12.6 ± 16.2	6.7	5.5 - 13.1
Small fish with bones (g)	7.8 ± 12.8	5.2	1.4 - 9.2	8.4 ± 13.1	5.3	0.0 - 10.2	7.4 ± 13.4	4.1	0.0 - 8.9
Canned tuna (g)	3.7 ± 5.5	2.8	0.6 - 4.6	3.7 ± 6.1	3.2	0.0 - 3.9	3.7 ± 5.5	2.8	0.0 - 3.7
Dried fish/salted fish (g)	22.2 ± 21.9	17.7	9.8 - 30.2	21.9 ± 25.1	13.2	6.8 - 33.0	22.4 ± 22.9	12.6	6.4 - 28.6
Oily fish (g)	19.7 ± 18.0	15.8	9.2 - 28.4	22.1 ± 21.4	15.2	7.1 - 34.2	18.0 ± 19.0	11.8	6.1 - 29.6
Lean fish (g)	20.2 ± 18.1	16.0	9.7 - 30.2	22.2 ± 21.8	15.2	7.8 - 34.2	18.8 ± 17.5	13.1	6.1 - 29.6
Raw fish (g)	24.2 ± 24.0	18.6	10.3 - 30.5	29.0 ± 29.4	20.0	10.6 - 36.5	20.7 ± 21.6	14.4	8.3 - 25.8
Grilled fish (g)	45.0 ± 35.8	37.6	21.1 - 59.8	44.9 ± 40.8	34.3	18.3 - 59.1	45.0 ± 36.6	36.7	18.5 - 60.0

Boiled fish (g)	52.4 ± 50.9	36.3	18.9 - 77.8	57.7 ± 59.1	35.5	16.6 - 87.5	48.5 ± 48.9	28.7	14.2 - 72.8
Fried fish (g)	18.6 ± 16.9	16.4	8.2 - 25.3	22.1 ± 20.1	17.7	8.3 - 31.3	16.0 ± 17.2	12.5	6.3 - 21.0
**Meat**									
Chicken (g)	25.8 ± 20.5	22.0	12.9 - 34.3	27.6 ± 23.1	17.1	12.8 - 35.6	24.5 ± 20.7	24.6	11.1 - 30.8
Pork/beef (g)	34.6 ± 21.7	32.6	20.1 - 39.7	37.4 ± 24.4	35.6	22.8 - 39.1	32.6 ± 21.6	30.8	14.8 - 33.9
Ham/sausage/bacon (g)	9.1 ± 8.1	7.4	4.0 - 12.3	10.0 ± 9.2	9.9	2.5 - 13.6	8.4 ± 8.2	4.7	2.2 - 10.7
Liver (g)	0.9 ± 2.0	0.2	-0.1 - 2.2	1.2 ± 2.2	0.0	0.0 - 2.8	0.7 ± 1.9	0.0	0.0 - 0.0
Grilled meat (g)	15.1 ± 18.0	11.3	4.0 - 19.0	17.7 ± 19.7	11.7	5.6 - 23.0	13.2 ± 17.6	9.0	0.0 - 16.1
Hamburg steak (g)	29.0 ± 24.4	22.3	14.6 - 37.7	32.2 ± 25.6	25.1	16.3 - 42.3	26.7 ± 23.8	19.0	13.1 - 34.1
Fried meat (g)	23.6 ± 19.4	18.9	11.3 - 30.2	27.2 ± 21.5	21.9	12.4 - 35.7	21.0 ± 19.1	15.3	9.6 - 26.7
Stir fry meat (g)	63.2 ± 41.5	58.0	33.3 - 82.6	61.5 ± 42.5	57.1	29.2 - 82.2	64.5 ± 42.4	57.0	33.6 - 83.2

Simmered meat (g)	99.7 ± 74.7	87.0	37.3 - 148.0	107.4 ± 82.8	90.6	38.2 - 161.3	94.2 ± 69.5	82.9	34.2 - 141.3
**Eggs**									
Egg (g)	40.0 ± 23.3	35.8	23.8 - 54.5	44.1 ± 29.3	29.9	24.5 - 59.9	37.0 ± 23.4	25.9	21.2 - 51.9
**Milk and dairy products**									
Low fat milk (g)	33.7 ± 63.1	3.3	-0.3 - 48.7	34.7 ± 68.3	0.0	0.0 - 27.2	32.9 ± 59.3	0.0	0.0 - 48.2
Milk (g)	69.5 ± 79.4	44.7	8.1 - 133.0	66.9 ± 90.3	22.3	0.0 - 123.7	71.4 ± 72.3	53.6	0.0 - 135.0
Ice cream (g)	18.1 ± 25.5	10.9	3.4 - 19.5	20.6 ± 30.0	9.2	0.0 - 19.8	16.3 ± 22.8	8.0	0.0 - 17.1
**Fats and oils**									
Cooking oil (g)	11.0 ± 4.8	10.6	7.7 - 13.8	11.8 ± 5.8	11.3	7.9 - 15.9	10.5 ± 4.9	9.8	7.0 - 13.4
**Confectioneries**									
Western-type confectioneries (g)	20.8 ± 21.9	15.0	7.1 - 28.6	19.7 ± 24.6	11.5	5.4 - 28.9	21.6 ± 22.0	10.0	4.7 - 25.0

Japanese-type confectioneries (g)	7.6 ± 9.5	5.2	2.3 - 9.1	6.6 ± 8.6	3.8	0.0 - 8.2	8.3 ± 10.7	3.3	3.3 - 7.1
Rice cracker/rice cake (g)	17.6 ± 15.2	15.0	7.8 - 23.4	18.3 ± 18.0	20.6	6.0 - 20.6	17.1 ± 15.5	17.9	7.1 - 17.9
**Alcoholic beverage**									
Sake (g)	12.5 ± 47.5	3.5	-3.5 - 10.8	26.6 ± 68.9	0.0	0.0 - 0.0	2.4 ± 20.8	0.0	0.0 - 0.0
Beer (g)	125 ± 257	49	5 - 139	220 ± 325	68	0 - 271	57 ± 180	0	0 - 16
Shochu (g)	19.0 ± 46.2	6.4	-2.1 - 16.8	35.4 ± 63.6	0.0	0.0 - 43.4	7.1 ± 26.5	0.0	0.0 - 0.0
Whiskey (g)	3.3 ± 16.1	0.7	-1.2 - 2.3	6.8 ± 23.6	0.0	0.0 - 0.0	0.7 ± 6.6	0.0	0.0 - 0.0
Wine (g)	6.0 ± 26.4	1.5	-2.3 - 5.0	8.8 ± 34.6	0.0	0.0 - 0.0	3.9 ± 19.6	0.0	0.0 - 0.0
**Non-alcoholic beverage**									
Green tea (g)	141 ± 170	69	20 - 168	134 ± 177	62	12 - 173	146 ± 168	107	10 - 150
Black tea/oolong tea (g)	43.4 ± 101.0	6.2	0.7 - 25.4	37.5 ± 93.8	0.0	0.0 - 24.7	47.6 ± 105.9	10.0	0.0 - 21.4
Coffee (g)	235 ± 180	170	90 - 392	258 ± 203	173	62 - 433	219 ± 163	150	107 - 375

Cola drink/soft drink (g)	73 ± 121	42	13 - 96	111 ± 160	82	15 - 165	45 ± 85	13	0 - 71
**Seasonings and spices**									
Mayonnaise/dressing (g)	6.2 ± 4.6	5.4	3.1 - 9.1	6.4 ± 5.3	5.8	2.3 - 10.4	6.0 ± 4.5	5.0	2.2 - 9.0
Miso soup (g)	165 ± 108	141	94 - 233	199 ± 132	152	125 - 277	141 ± 99	108	60 - 216
Noodle soup (g)	94 ± 70	79	50 - 122	124 ± 93	97	59 - 165	72 ± 59	56	34 - 94
Soy sauce (g)	1.6 ± 0.4	1.6	1.3 - 1.9	1.9 ± 0.5	1.8	1.5 - 2.2	1.4 ± 0.4	1.4	1.2 - 1.6
Cooking salt (g)	3.4 ± 1.0	3.5	2.8 - 4.0	3.7 ± 1.3	3.6	2.8 - 4.4	3.2 ± 1.1	3.2	2.5 - 3.8

SFA; saturated fatty acid, MUFA; mono-unsaturated fatty acid, PUFA; poly-unsaturated fatty acid, eq.; equivalent

Table S4. Relative abundance (%) of each gut bacterium of the participants.

Variable	All subjects			Male			Female		
	Mean ± SD	Median	IQR	Mean ± SD	Median	IQR	Mean ± SD	Median	IQR
phylum Actinobacteria	13.3 ± 10.0	10.8	5.9 - 18.2	13.4 ± 10.3	10.8	6.1 - 18.0	13.3 ± 9.8	10.9	5.7 - 18.3
class Actinobacteria	13.3 ± 10.0	10.8	5.9 - 18.2	13.4 ± 10.3	10.8	6.1 - 18.0	13.3 ± 9.8	10.9	5.7 - 18.3
order Bifidobacteriales	7.6 ± 8.2	5.0	1.5 - 11.0	7.3 ± 8.6	4.2	1.4 - 10.2	7.8 ± 7.9	5.5	1.5 - 11.6
family Bifidobacteriaceae	7.6 ± 8.2	5.0	1.5 - 11.0	7.3 ± 8.6	4.2	1.4 - 10.2	7.8 ± 7.9	5.5	1.5 - 11.6
genus Bifidobacterium	7.6 ± 8.2	5.0	1.5 - 11.0	7.3 ± 8.6	4.2	1.4 - 10.2	7.8 ± 7.9	5.5	1.5 - 11.6
class Coriobacteriia	NA	NA	NA	NA	NA	NA	NA	NA	NA
order Coriobacteriales	5.7 ± 4.9	5.0	1.3 - 8.5	6.1 ± 4.7	5.7	2.1 - 8.7	5.3 ± 4.9	4.3	1.1 - 8.1
family Coriobacteriaceae	5.7 ± 4.9	5.0	1.3 - 8.5	6.1 ± 4.7	5.7	2.1 - 8.7	5.3 ± 4.9	4.3	1.1 - 8.1
genus Collinsella	4.9 ± 4.7	4.2	0.5 - 7.6	5.4 ± 4.5	5.0	1.6 - 8.0	4.6 ± 4.8	3.8	0.0 - 7.2
phylum Bacteroidetes	21.1 ± 10.2	19.0	14.2 - 26.8	22.6 ± 11.3	20.4	15.4 - 29.4	20.0 ± 9.3	18.3	13.9 - 24.6
class Bacteroidia	21.0 ± 10.3	19.0	14.2 - 26.8	22.5 ± 11.3	20.4	15.4 - 29.3	20.0 ± 9.3	18.2	13.8 - 24.6
order Bacteroidales	21.0 ± 10.3	19.0	14.2 - 26.8	22.5 ± 11.3	20.4	15.4 - 29.3	20.0 ± 9.3	18.2	13.8 - 24.6
family Prevotellaceae	6.5 ± 11.4	0.1	0.0 - 8.4	9.2 ± 13.3	0.9	0.0 - 16.8	4.6 ± 9.4	0.0	0.0 - 3.3

genus Prevotella	5.9 ± 10.9	0.0	0.0 - 7.3	8.4 ± 12.7	0.0	0.0 - 15.4	4.1 ± 9.1	0.0	0.0 - 2.6
family Bacteroidaceae	10.9 ± 7.2	10.0	5.1 - 15.3	10.5 ± 7.3	9.3	4.7 - 15.3	11.2 ± 7.2	10.4	5.4 - 15.3
genus Bacteroides	10.9 ± 7.2	10.0	5.1 - 15.3	10.5 ± 7.3	9.3	4.7 - 15.3	11.2 ± 7.2	10.4	5.4 - 15.3
family Rikenellaceae	2.1 ± 2.8	1.1	0.1 - 2.9	1.4 ± 2.1	0.5	0.1 - 1.9	2.6 ± 3.1	1.6	0.3 - 3.7
genus Alistipes	2.1 ± 2.8	1.1	0.1 - 2.9	1.4 ± 2.0	0.5	0.1 - 1.9	2.6 ± 3.1	1.6	0.3 - 3.7
family Porphyromonadaceae	1.5 ± 1.6	1.1	0.5 - 1.9	1.4 ± 1.7	0.9	0.4 - 1.8	1.5 ± 1.5	1.1	0.6 - 2.0
genus Parabacteroides	1.0 ± 1.3	0.6	0.3 - 1.2	1.0 ± 1.5	0.5	0.2 - 1.2	1.0 ± 1.2	0.7	0.3 - 1.3
phylum Firmicutes	61.6 ± 12.0	62.6	54.2 - 70.5	59.3 ± 12.8	60.3	51.1 - 68.7	63.4 ± 11.1	64.3	56.4 - 71.2
class Clostridia	53.1 ± 14.4	54.6	43.9 - 63.7	49.5 ± 15.5	51.1	39.3 - 60.9	55.6 ± 13.0	57.3	47.4 - 64.7
order Clostridiales	53.1 ± 14.4	54.6	43.9 - 63.7	49.5 ± 15.5	51.1	39.3 - 60.9	55.6 ± 13.0	57.3	47.4 - 64.7
family Lachnospiraceae	32.4 ± 11.7	31.5	24.3 - 40.1	32.4 ± 12.1	31.3	24.4 - 40.3	32.4 ± 11.5	31.7	24.3 - 40.0
genus Blautia	7.3 ± 4.2	6.6	4.5 - 9.1	6.9 ± 3.9	6.3	4.4 - 8.8	7.5 ± 4.4	6.8	4.6 - 9.4
genus Anaerostipes	5.0 ± 5.5	3.4	1.1 - 7.1	5.3 ± 6.0	3.3	1.1 - 7.2	4.9 ± 5.1	3.4	1.0 - 7.0
genus Roseburia	4.0 ± 3.8	3.1	1.2 - 5.8	4.2 ± 3.9	3.2	1.3 - 6.0	3.9 ± 3.7	3.0	1.1 - 5.7
genus Fusicatenibacter	2.4 ± 2.4	1.9	0.5 - 3.5	2.3 ± 2.2	1.9	0.5 - 3.3	2.5 ± 2.5	1.9	0.5 - 3.6

genus Lachnospiracea incertae sedis	1.9 ± 1.3	1.7	1.0 - 2.5	1.6 ± 1.1	1.4	0.9 - 2.2	2.0 ± 1.4	1.8	1.1 - 2.7
family Ruminococcaceae	19.3 ± 11.3	18.9	10.1 - 27.5	15.8 ± 10.9	15.2	6.5 - 23.3	21.8 ± 11.0	21.3	13.1 - 29.7
genus Faecalibacterium	7.7 ± 5.6	7.1	3.3 - 11.1	6.6 ± 5.3	6.1	2.5 - 9.5	8.4 ± 5.6	7.9	4.1 - 11.8
genus Ruminococcus	3.3 ± 4.5	1.0	0.0 - 5.6	2.6 ± 3.7	0.5	0.0 - 4.4	3.9 ± 4.9	1.9	0.0 - 6.4
genus Ruminococcus2	5.4 ± 5.6	3.9	1.3 - 7.6	5.4 ± 5.6	3.9	1.0 - 7.6	5.5 ± 5.6	4.0	1.4 - 7.5
genus Gemmiger	2.4 ± 2.5	1.9	0.4 - 3.7	2.1 ± 2.2	1.6	0.1 - 3.1	2.7 ± 2.6	2.2	0.7 - 4.0
family Clostridiaceae	NA	NA	NA	NA	NA	NA	NA	NA	NA
genus Clostridium IV	2.4 ± 3.8	0.9	0.3 - 2.7	1.8 ± 3.2	0.5	0.2 - 2.1	2.8 ± 4.2	1.3	0.4 - 3.4
class Negativicutes	3.1 ± 4.3	1.6	0.9 - 3.6	3.9 ± 5.1	2.2	1.2 - 4.6	2.5 ± 3.6	1.4	0.8 - 2.6
order Selenomonadales	3.1 ± 4.3	1.6	0.9 - 3.6	3.9 ± 5.1	2.2	1.2 - 4.6	2.5 ± 3.6	1.4	0.8 - 2.6
family Veillonellaceae	2.3 ± 4.3	0.9	0.2 - 2.6	2.9 ± 5.1	1.1	0.2 - 3.4	1.9 ± 3.5	0.8	0.1 - 2.1
genus Megamonas	1.0 ± 3.8	0.0	0.0 - 0.0	1.5 ± 4.7	0.0	0.0 - 0.0	0.7 ± 3.0	0.0	0.0 - 0.0
class Erysipelotrichia	2.7 ± 2.9	1.7	0.7 - 3.8	2.9 ± 3.1	1.9	0.7 - 4.1	2.6 ± 2.8	1.7	0.7 - 3.5
order Erysipelotrichales	2.7 ± 2.9	1.7	0.7 - 3.8	2.9 ± 3.1	1.9	0.7 - 4.1	2.6 ± 2.8	1.7	0.7 - 3.5

family Erysipelotrichaceae	2.7 ± 2.9	1.7	0.7 - 3.8	2.9 ± 3.1	1.9	0.7 - 4.1	2.6 ± 2.8	1.7	0.7 - 3.5
class Bacilli	2.6 ± 4.5	1.1	0.4 - 2.7	2.9 ± 4.8	1.1	0.4 - 2.8	2.5 ± 4.3	1.1	0.4 - 2.7
order Lactobacillales	2.6 ± 4.5	1.0	0.4 - 2.6	2.8 ± 4.8	1.0	0.3 - 2.6	2.4 ± 4.3	1.0	0.4 - 2.5
family Streptococcaceae	2.1 ± 3.7	0.8	0.3 - 2.1	2.1 ± 3.5	0.8	0.3 - 2.0	2.0 ± 3.8	0.8	0.3 - 2.1
genus Streptococcus	2.0 ± 3.7	0.8	0.3 - 2.1	2.1 ± 3.5	0.8	0.3 - 2.0	2.0 ± 3.8	0.8	0.3 - 2.1
phylum Proteobacteria	2.6 ± 2.8	2.0	1.2 - 3.2	3.1 ± 3.2	2.3	1.4 - 3.6	2.3 ± 2.4	1.7	1.0 - 2.8
class Betaproteobacteria	1.5 ± 1.3	1.3	0.5 - 2.1	1.8 ± 1.5	1.5	0.7 - 2.5	1.3 ± 1.1	1.1	0.5 - 1.9
order Burkholderiales	1.5 ± 1.3	1.3	0.5 - 2.1	1.8 ± 1.5	1.5	0.7 - 2.5	1.3 ± 1.1	1.1	0.5 - 1.9
family Sutterellaceae	1.4 ± 1.3	1.1	0.3 - 2.0	1.7 ± 1.5	1.4	0.5 - 2.4	1.2 ± 1.2	0.9	0.1 - 1.7
family Unclassified	1.0 ± 1.5	0.5	0.2 - 1.1	0.8 ± 1.3	0.4	0.1 - 0.8	1.1 ± 1.5	0.6	0.2 - 1.3
genus Unclassified	8.3 ± 4.7	7.3	5.1 - 10.1	7.9 ± 4.7	6.7	4.9 - 9.4	8.6 ± 4.7	7.6	5.3 - 10.4

NA; not analyzed

Table S5. Association between plasma LBP concentration and clinical markers or clinical scores[1] (All data)

Category	Variable	*β*	*Q* value
Obesity	Abdominal circumference (cm)[2]	0.02	0.05*
	Body fat percentage (%)	0.03	0.32
	Body water content (kg)	-0.02	0.21
	Visceral fat level (levels)	0.09	0.03*
	Basal metabolic rate (kcal/day)	-0.01	0.53
	Basal metabolic rate level (levels)	-0.08	0.03*
Blood pressure	Systolic blood pressure (mmHg)	0.07	<0.001*
	Diastolic blood pressure (mmHg)	0.05	0.05*
Arteriosclerosis	baPWV (cm/s)	0.08	<0.001*
Glucose metabolism	HOMA-IR	0.18	0.04*
	Blood glucose (mg/dL)	0.02	0.41
	HbA1c (%)	0.04	0.009*
	Blood insulin (μU/mL)	0.16	0.04*
	C peptide (ng/mL)	0.14	0.009*
	Glycoalbumin (%)	0.02	0.37
Lipid metabolism	Triglyceride (mg/dL)	0.13	0.14*
	Total cholesterol (mg/dL)	0.01	0.63
	HDL-cholesterol (mg/dL)	-0.12	<0.001*
	LDL-cholesterol (mg/dL)	0.04	0.40
Physical function	Estimated bone mass (kg)	-0.01	0.63
	Muscle mass (kg)	-0.01	0.52
	Right leg (kg)	-0.02	0.27
	Left leg (kg)	-0.02	0.29
	Right arm (kg)	-0.02	0.19*
	Left arm (kg)	-0.03	0.13*
	Trunk (kg)	0.00	0.80

	Locomo 25 score (points)	-1.84	0.24
Eyesight	Far-sightedness	-0.05	0.49
	Near-sightedness	-0.02	0.73
Liver function	Albumin (g/dL)	-0.01	0.16*
	AST (U/L)	0.16	<0.001*
	ALT (U/L)	0.17	0.02*
	γ-GTP (U/L)	0.31	<0.001*
	Total bilirubin (mg/dL)	-0.11	0.07*
	Total Protein (g/dL)	0.03	<0.001*
Renal function	Creatinine (mg/dL)	0.02	0.48
	Blood urea nitrogen (mg/dL)	-0.01	0.75
	Plasma renin activity (ng/mL/hr)	0.27	0.16*
	Urine albumin creatinine ratio (mg/gCr)	0.50	0.004*
Adrenal cortex function	Aldosterone (pg/mL)	-0.01	0.77
	Cortisol (μg/dL)	0.14	0.02*
Cardiac function	Brain natriuretic peptide (pg/mL)	0.11	0.31
	Troponin I (pg/mL)	0.01	0.76
Thyroid function	Free thyroxine (ng/dL)	0.05	0.02*
	Thyroid stimulating hormone (μIU/mL)	-0.07	0.58
Sexual function	Testosterone (ng/mL)	-0.08	0.26
Inflammation	Neutrophil (%)	0.11	<0.001*
	Stab neutrophil (%)	0.44	<0.001*
	Segmented neutrophil (%)	0.09	<0.001*
	Lymphocyte (%)	-0.16	<0.001*
	Monocyte (%)	0.09	0.20
	Eosinophil (%)	-0.67	0.06*
	Basophil (%)	-0.18	0.10*
	IgG (mg/dL)	0.06	0.10*
	IgA (mg/dL)	0.17	0.009*
	IgM (mg/dL)	0.06	0.52

	Complement C3 (mg/dL)	0.16	<0.001*
	Complement C4 (mg/dL)	0.25	<0.001*
	hs-CRP (mg/dL)	1.52	<0.001*
	IL-6 (pg/mL)	0.43	<0.001*
Oxidative stress	8-OHdG (ng/mL)	-0.08	0.29
Hematological test	White blood cell (cells/μL)	0.16	<0.001*
	Red blood cell (10^4 cells/μL)	-0.01	0.54
	Hemoglobin (g/dL)	-0.01	0.54
	Hematocrit (%)	-0.54	0.32
	Mean cell volume (fL)	0.00	0.60
	Mean corpuscular hemoglobin (pg)	0.00	0.80
	Mean cell hemoglobin concentration (g/dL)	0.00	0.46
	Platelet (10^4 cells/μL)	0.05	0.23
Iron metabolism	Ferritin (ng/mL)	-0.02	0.77
	Serum iron (mmol/L)	-0.14	0.07*
Electrolyte	Blood sodium (mmol/L)	0.00	0.24
	Blood potassium (mmol/L)	0.00	0.76
Allergy	Total IgE (IU/mL)	0.01	0.79
	Anti-poaceae pollen IgE (UA/mL)	-0.23	0.41
	Anti-Weed IgE (UA/mL)	-0.10	0.56
	Anti-House dust IgE (UA/mL)	-0.12	0.63
	Anti-Cedar pollen IgE (UA/mL)	-0.19	0.57
Rheumatoid arthritis	Anti-CCP antibody (U/mL)	0.09	0.52
Pituitary gland function	Prolactin (ng/mL)	0.03	0.68
	Follicle stimulating hormone (mlU/mL)	0.02	0.76
	Adrenocorticotropic hormone (pg/mL)	0.05	0.57
Asthma/airway inflammation	FVC (L)	0.00	0.78
	FVC to predicted FVC ratio (%)	-0.01	0.72
	FEV1 (L)	0.00	0.78

	FEV1 to predicted FEV1 ratio (%)	0.00	0.8
	FEV1%G (%)	0.01	0.67
	FEV1%G to predicted FEV1%G ratio (%)	0.01	0.60
	MMF (L/s)	0.04	0.49
	MMF to predicted MMF ratio (%)	0.04	0.52
	PEF (L/s)	-0.02	0.64
	PEF to predicted PEF ratio (%)	-0.02	0.62
	PEF time (seconds)	0.04	0.58
	FET (seconds)	0.10	0.34
	EV (%)	0.02	0.70
	Exhaled H_2 (ppm)	-0.07	0.67
	Exhaled CH_4 (ppm)	-0.03	0.72
Mental health	S-WHO-5 score (points)	0.05	0.78
Depression	CES-D score (points)	-0.69	0.61
Cognitive function	MMSE score (points)	0.09	0.63

*$Q < 0.20$

[1] Multiple liner regression model was adjusted for age, sex, body mass index, smoking habit (current habitual smoker or not), drinking habit (current habitual drinker or not).

[2] The units used for the calculation of the β values are indicated.

baPWV, brachial-ankle pulse wave velocity; HOMA-IR, homeostasis model assessment-insulin resistance; HbA1c, Hemoglobin A1c; HDL, high-density lipoprotein; LDL, low-density lipoprotein; AST, aspartate aminotransferase; ALT, alanine aminotransferase; γ-GTP, γ-glutamyltransferase; Ig, immunoglobulin; hs-CRP, high-sensitivity C-reactive protein; IL, interleukin; 8-OHdG, 8-hydroxy-2-deoxyguanosine; CCP, cyclic citrullinated peptide; FVC, forced vital capacity; FEV, forced expiratory volume in one second; FEV1%G; FEV1/FVC; MMF, maximum mid-expiratory flow; PEF, peak expiratory flow; FET, Forced expiration time; EV, extrapolated volume; S-WHO-5, simplified Japanese version of the World Health Organization-Five Well-Being Index; CES-D, Center for Epidemiological Studies Depression; MMSE, Mini-Mental state examination

Table S6. Association between plasma LBP concentration and dietary factors[1,2] (All data)

Category	Variable	*β*	*Q* value
Blood antioxidant	Total carotenoids (μg/mL)[3]	-0.05	0.19*
	Lutein (μg/mL)	-0.04	0.33
	Zeaxanthin (μg/mL)	-0.02	0.55
	β-Cryptoxanthin (μg/mL)	-0.05	0.12*
	α-Carotene (μg/mL)	-0.01	0.71
	β-Carotene (μg/mL)	-0.04	0.08*
	Lycopene (μg/mL)	-0.02	0.40
	Retinol (μg/mL)	0.09	0.08*
	Vitamin C (μg/mL)	0.06	0.16*
	α-Tocopherol (μg/mL)	0.06	0.36
Nutrition intake	Protein (100 g)	-0.11	0.36
	Fat (100 g)	-0.19	0.15*
	SFA (100 g)	-0.48	0.26
	MUFA (100 g)	-0.47	0.17*
	PUFA (100 g)	-0.67	0.19*
	Cholesterol (100 mg)	-0.01	0.43
	Carbohydrate (100 g)	0.00	0.80
	Total dietary fiber (100 g)	-0.75	0.11*
	Soluble (100 g)	-2.62	0.10*
	Insoluble (100 g)	-1.09	0.11*
	Sodium (100 mg)	0.00	0.69
	Potassium (100 mg)	-0.004	0.17*
	Calcium (100 mg)	-0.01	0.42
	Magnesium (100 mg)	-0.03	0.31
	Phosphorus (100 mg)	-0.01	0.36

	Iron (100 mg)	-0.86	0.32
	Zinc (100 mg)	-1.63	0.21
	Copper (100 mg)	-7.82	0.37
	Manganese (100 mg)	2.64	0.22
	Retinol eq. (100 μg RE)	0.00	0.79
	Vitamin D (100 μg)	0.01	0.79
	α-Tocopherol (100 mg)	-1.05	0.22
	Vitamin K (100 μg)	-0.02	0.16*
	Vitamin B_1 (100 mg)	-13.90	0.13*
	Vitamin B_2 (100 mg)	-3.41	0.51
	Niacin (100 mg)	-0.22	0.49
	Vitamin B_6 (100 mg)	-5.37	0.28
	Vitamin B_{12} (100 μg)	0.07	0.72
	Folate (100 μg)	-0.01	0.49
	Pantothenic acid (100 mg)	-1.83	0.14*
	Alcohol (100 g)	0.14	0.12*
Food intake			
Cereals	Bread (100 g)	0.01	0.72
	Buckwheat noodles (100 g)	0.04	0.52
	Japanese wheat noodles (100 g)	0.06	0.44
	Chinese noodles (100 g)	0.06	0.30
	Spaghetti and macaroni (100 g)	-0.07	0.54
	Rice (100 g)	0.00	0.76
Potatoes	Potatoes (100 g)	-0.05	0.38
Sugars and sweeteners	Sugar (100 g)	0.07	0.74
	Cooking sugar (100 g)	-0.40	0.60
Pulses	Tofu/Deep fried tofu (100 g)	-0.03	0.49
	Natto (100 g)	-0.07	0.45

Vegetables	Pickled green leaves vegetables (100 g)	-0.09	0.58
	Other pickled vegetables (100 g)	-0.09	0.54
	Raw lettuces/cabbage (100 g)	-0.10	0.22
	Green leaves vegetables (100 g)	-0.03	0.52
	Cabbage/Chinese cabbage (100 g)	-0.10	0.11*
	Carrots/pumpkin (100 g)	-0.10	0.39
	Japanese radish/turnip (100 g)	-0.14	0.12*
	Other root vegetables (100 g)	-0.07	0.30
	Tomatoes (100 g)	-0.10	0.09*
Fruits	Citrus fruit (100 g)	-0.05	0.53
	Persimmons/strawberries/kiwifruit (100 g)	0.00	0.80
	Other fruit (100 g)	0.01	0.77
	100% fruit and vegetable juice (100 g)	0.01	0.60
	Seasonal citrus (100 g)	-0.05	0.66
	Seasonal persimmon (100 g)	-0.16	0.31
	Seasonal strawberry (100 g)	0.02	0.78
Mushroom	Mushrooms (100 g)	-0.05	0.71
Algae	Seaweeds (100 g)	-0.06	0.64
Fish and shellfish	Squid/octopus/shrimp/shellfish (100 g)	0.10	0.32
	Small fish with bones (100 g)	0.06	0.60
	Canned tuna (100 g)	-0.15	0.56
	Dried fish/salted fish (100 g)	0.00	0.80
	Oily fish (100 g)	-0.02	0.72
	Lean fish (100 g)	0.01	0.78
	Raw fish (100 g)	0.11	0.08*
	Grilled fish (100 g)	-0.03	0.51
	Boiled fish (100 g)	0.00	0.80
	Fried fish (100 g)	0.06	0.49
Meat	Chicken (100 g)	-0.09	0.21
	Pork/beef (100 g)	-0.04	0.58

	Ham/sausage/bacon (100 g)	-0.07	0.65
	Liver (100 g)	0.59	0.44
	Grilled meat (100 g)	0.01	0.79
	Hamburg steak (100 g)	-0.03	0.59
	Fried meat (100 g)	-0.03	0.66
	Stir fry meat (100 g)	0.00	0.77
	Simmered meat (100 g)	-0.01	0.55
Eggs	Egg (100 g)	-0.05	0.42
Milk and dairy products	Low fat milk (100 g)	0.00	0.80
	Milk (100 g)	-0.01	0.48
	Ice cream (100 g)	0.03	0.59
Fats and oils	Cooking oil (100 g)	-0.04	0.78
Confectioneries	Western-type confectioneries (100 g)	-0.06	0.43
	Japanese-type confectioneries (100 g)	-0.10	0.54
	Rice cracker/rice cake (100 g)	0.00	0.80
Alcoholic beverage	Sake (100 g)	0.00	0.79
	Beer (100 g)	0.00	0.56
	Shochu (100 g)	0.05	0.11*
	Whiskey (100 g)	0.05	0.55
	Wine (100 g)	0.03	0.54
Non-alcoholic beverage	Green tea (100 g)	0.01	0.13*
	Black tea/oolong tea (100 g)	0.02	0.31
	Coffee (100 g)	-0.01	0.51
	Cola drink/soft drink (100 g)	0.00	0.79
Seasonings and spices	Mayonnaise/dressing (100 g)	-0.39	0.23
	Miso soup (100 g)	-0.01	0.31
	Noodle soup (100 g)	0.02	0.32
	Soy sauce (100 g)	-2.91	0.44
	Cooking salt (100 g)	-0.65	0.64

*$Q < 0.20$

[1] Multiple liner regression model was adjusted for age, sex, body mass index, smoking habit (current habitual smoker or not), drinking habit (current habitual drinker or not), and energy intake.
[2] Nutrition intake and food intake were adjusted by energy intake using residual method.
[3] The units used for the calculation of the β values are indicated.
SFA; saturated fatty acid, MUFA; mono-unsaturated fatty acid, PUFA; poly-unsaturated fatty acid, eq.; equivalent

References

[130] H. Hamamura, H. Adachi, M. Enomoto, A. Fukami, S. Nakamura, Y. Nohara, N. Morikawa, A. Sakaue, K. Toyomasu, M. Yamamoto, Y. Fukumoto, Serum Proprotein Convertase Subtilisin/Kexin Type 9 (PCSK9) is Independently Associated with Insulin Resistance, Triglycerides, Lipoprotein(a) Levels but not Low-Density Lipoprotein Cholesterol Levels in a General Population, J. Atheroscler. Thromb. 28 (2021) 329–337. https://doi.org/10.5551/jat.56390.

[131] Y. Hoshino, A. Seichi, [Locomo 25--a screening tool for risk of locomotive syndrome]., Nihon Rinsho. 72 (2014) 1839–43. http://www.ncbi.nlm.nih.gov/pubmed/25509812.

[132] K. Ikeda, K. Noda, S. Yamaguchi, A Relation between Adaptation Luminance and Visual Acuity for the Landort Ring under the Uniform Background, J. Illum. Eng. Inst. Japan. 64 (1980) 591–597. https://doi.org/10.2150/jieij1980.64.10_591.

[133] H. Inagaki, K. Ito, N. Sakuma, M. Sugiyama, T. Okamura, S. Awata, [Reliability and validity of the simplified Japanese version of the WHO-Five Well-being Index (S-WHO-5-J)]., Nihon. Koshu Eisei Zasshi. 60 (2013) 294–301. http://www.ncbi.nlm.nih.gov/pubmed/23942026.

[134] L.S. Radloff, The CES-D Scale, Appl. Psychol. Meas. 1 (1977) 385–401. https://doi.org/10.1177/014662167700100306.

[135] M.F. Folstein, S.E. Folstein, P.R. McHugh, “Mini-mental state”. A practical method for grading the cognitive state of patients for the clinician., J. Psychiatr. Res. 12 (1975) 189–98. https://doi.org/10.1016/0022-3956(75)90026-6.
